# Quantifying dysmorphologies of the neurocranium using artificial neural networks

**DOI:** 10.1111/joa.14061

**Published:** 2024-05-17

**Authors:** Tareq Abdel‐Alim, Franz Tapia Chaca, Irene M. J. Mathijssen, Clemens M. F. Dirven, Wiro J. Niessen, Eppo B. Wolvius, Marie‐Lise C. van Veelen, Gennady V. Roshchupkin

**Affiliations:** ^1^ Department of Neurosurgery Erasmus Medical Center Rotterdam The Netherlands; ^2^ Department of Radiology and Nuclear Medicine Erasmus Medical Center Rotterdam The Netherlands; ^3^ Department of Plastic and Reconstructive Surgery Erasmus Medical Center Rotterdam The Netherlands; ^4^ Faculty of Medical Sciences University of Groningen Groningen The Netherlands; ^5^ Department of Oral‐ and Maxillofacial Surgery Erasmus Medical Center Rotterdam The Netherlands; ^6^ Department of Epidemiology Erasmus Medical Center Rotterdam The Netherlands

**Keywords:** artificial intelligence, craniofacial dysmorphologies, craniosynostosis, neural networks, photogrammetry, quantification, quantitative morphometry, shape analysis

## Abstract

**Background:**

Craniosynostosis, a congenital condition characterized by the premature fusion of cranial sutures, necessitates objective methods for evaluating cranial morphology to enhance patient treatment. Current subjective assessments often lead to inconsistent outcomes. This study introduces a novel, quantitative approach to classify craniosynostosis and measure its severity.

**Methods:**

An artificial neural network was trained to classify normocephalic, trigonocephalic, and scaphocephalic head shapes based on a publicly available dataset of synthetic 3D head models. Each 3D model was converted into a low‐dimensional shape representation based on the distribution of normal vectors, which served as the input for the neural network, ensuring complete patient anonymity and invariance to geometric size and orientation. Explainable AI methods were utilized to highlight significant features when making predictions. Additionally, the Feature Prominence (FP) score was introduced, a novel metric that captures the prominence of distinct shape characteristics associated with a given class. Its relationship with clinical severity scores was examined using the Spearman Rank Correlation Coefficient.

**Results:**

The final model achieved excellent test accuracy in classifying the different cranial shapes from their low‐dimensional representation. Attention maps indicated that the network's attention was predominantly directed toward the parietal and temporal regions, as well as toward the region signifying vertex depression in scaphocephaly. In trigonocephaly, features around the temples were most pronounced. The FP score showed a strong positive monotonic relationship with clinical severity scores in both scaphocephalic (*ρ* = 0.83, *p* < 0.001) and trigonocephalic (*ρ* = 0.64, *p* < 0.001) models. Visual assessments further confirmed that as FP values rose, phenotypic severity became increasingly evident.

**Conclusion:**

This study presents an innovative and accessible AI‐based method for quantifying cranial shape that mitigates the need for adjustments due to age‐specific size variations or differences in the spatial orientation of the 3D images, while ensuring complete patient privacy. The proposed FP score strongly correlates with clinical severity scores and has the potential to aid in clinical decision‐making and facilitate multi‐center collaborations. Future work will focus on validating the model with larger patient datasets and exploring the potential of the FP score for broader applications. The publicly available source code facilitates easy implementation, aiming to advance craniofacial care and research.

## Graphical abstract

This study introduces a novel explainable AI approach for the objective quantification of craniosynostosis severity, based on a reduced shape representation. This ensures complete patient anonymity and invariance to geometric size and orientation. The proposed severity metric, the “Feature Prominence Score,” captures distinct shape characteristics associated with specific classes and strongly correlates with clinical severity scores, providing a metric for objective treatment evaluation.

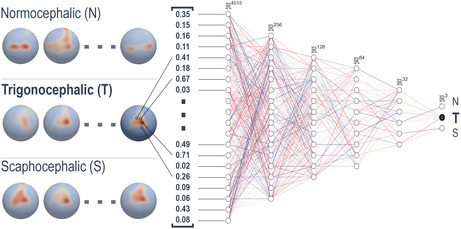

## 1 INTRODUCTION

Craniosynostosis, a congenital condition characterized by the premature fusion of cranial sutures, impacts both the functional aspects of brain development and the cranial morphology (Ghali et al., 2002; Kabbani & Raghuveer, 2004; van Veelen‐Vincent et al., 2010). To evaluate, compare, and ultimately enhance patient treatment, we need to be able to quantify the severity of a dysmorphology. Historically, severity assessments have been inherently subjective, relying on visual evaluations and 2D photo scores (Gaillard, 2023a, 2023b; Wes et al., 2017). This subjectivity potentially leads to varied treatment outcomes and can cloud postoperative evaluations and interpretations of published results. To surmount these challenges, there has been a long‐standing need for objective metrics that can capture the intricate details of cranial dysmorphologies in three dimensions and, in turn, evaluate and guide clinical interventions.

Three‐dimensional (3D) photogrammetry offers a non‐invasive and radiation‐free approach to acquire detailed surface reconstructions of a patient's head. Its ability to rapidly gather high‐dimensional data in a safe manner provides researchers with a powerful tool for longitudinal analysis. Use cases of this 3D data have not only deepened our understanding of craniofacial anomalies and their related phenotypes but also highlight its rising prominence in both future research and clinical practice (Abdel‐Alim et al., 2021).

In recent years, some research efforts have been directed toward the development of severity metrics and specific phenotypic indices for craniosynostosis (Anstadt et al., 2023; Bhalodia et al., 2020; Bins, Cull, et al., 2023; Bins, Zhou, et al., 2023; Elkhill et al., 2023; Liaw et al., 2019). In the same time frame, several studies have highlighted the potential of Artificial Intelligence (AI) in distinguishing craniosynostosis subtypes based on 3D data (Cho et al., 2018; de Jong et al., 2020; Geisler et al., 2021; Mashouri et al., 2020; Mizutani et al., 2022; Schaufelberger et al., 2023; Schaufelberger, Kühle, Kaiser, et al., 2022). While such pioneering studies are crucial for advancing the field and showcasing the technical capabilities of AI methods in comprehending 3D forms, merely differentiating between craniosynostosis subtypes offers limited clinical significance.

Considering the remarkable progress in healthcare AI and explainable AI over the past decade, and the anticipated integration of this technology into everyday clinical practice, it is rather surprising that only a handful of studies have explored the 3D assessment of craniosynostosis severity using AI techniques (Topol, 2019; Yu et al., 2018). Often, sample sizes in 3D studies focusing on craniosynostosis are limited, potentially hindering the broader adoption of data‐driven approaches. This emphasizes the importance of multi‐center collaborations. By pooling data from multiple centers, researchers can build larger and more representative datasets, which are crucial for training robust AI models. However, such collaborations present challenges. Sharing patient data across institutions raises significant data privacy concerns and still often forms a bottleneck in collaborative efforts. Even when collaborations are initiated, variations in data processing protocols, perhaps stemming from the absence or inconsistency of standard procedures, along with differences in the ages at which images are captured, can contribute to unbalanced datasets.

A potential solution lies in the separation of ‘shape’ as a distinct feature from a 3D image, mitigating the need for adjustments due to age‐specific size variations or differences in spatial orientation.

Over a decade ago, Atmosukarto et al (Atmosukarto et al., 2010). proposed a shape analysis method based on surface normal vectors in a craniofacial context using 3D images, specifically to quantify the amount of asymmetric flattening of the occiput in children with deformational plagiocephaly. A ‘flatness score’ was derived by counting the number of unidirectional vectors within predetermined bins. However, this method is unable to capture phenotypic variations beyond mere flatness as it does not consider underlying patterns and relationships between areas of high and low vector density, which is essential for assessing the overall morphology and intricate phenotypes. A few years later, an elegant improvement on this method was proposed by Vuollo et al (2016). Instead of confining the data to fixed bins, a smooth kernel density estimate (KDE) was used. However, this method was never extended beyond the quantification of cranial flatness and asymmetry.

In this study, we continue to build upon the ideas and principles previously discussed, integrating artificial neural networks to maximize insights from the surface normal density distribution. Our findings demonstrated that this distribution embeds comprehensive morphological information, allowing not just the differentiation between craniosynostosis subtypes, but also quantification of the phenotypic severity. This led to the introduction of the Feature Prominence (FP) score, a novel metric that captures the prominence of distinct shape characteristics associated with a given class. The FP score is defined by the raw output value of our neural network prior to classification, representing an integrated measure of cranial abnormality solely based on kernel density estimation values. The absolute FP score value in itself does not have a direct interpretive meaning. However, when normalized in relation to the morphological dataset used for training, it becomes a meaningful quantifiable index that reflects deviations in cranial shape associated with craniosynostosis severity.

The FP score is distinct from traditional linear measurements in that it provides a comprehensive and holistic evaluation of cranial shapes by interpreting the entirety of the three‐dimensional structure. This is crucial as the pathological severity in conditions like craniosynostosis is not solely determined by linear dimensions such as head circumference, cephalic index, or the bifrontal angle. Instead, the extent of the abnormality involves a more complex interplay of ratios and overall cranial shape characteristics, and therefore requires a holistic metric that captures these intricacies.

## 2 METHODS

### 2.1 Cranial data collection and pre‐processing

Synthetic 3D head models used for training, validation, and testing were extracted from a publicly available craniosynostosis dataset. This dataset was generated using a statistical shape model based on children younger than 1.5 years; the age distribution is detailed in the original paper (Schaufelberger, Kühle, Wachter, et al., 2022). The extracted data contained a total of 300 head models, comprising normocephalic (*n* = 100), trigonocephalic (*n* = 100), and scaphocephalic (*n* = 100) meshes.

During pre‐processing, the data were clipped along the nasion‐tragus plane and a template mesh was deformed to match the shape of each 3D surface using a variant of the non‐rigid iterative closest point algorithm (Amberg et al., 2007). This process preserves the topology of the template and guarantees consistent point correspondences throughout the entire collection of 3D data samples.

### 2.2 Normal density sphere

The Normal Density Sphere (NDS) is a lower‐dimensional shape representation that leverages the distribution of normal vectors on the 3D surface. Atmosukarto et al. recognized that a flat region is characterized by a greater concentration of unidirectional normal vectors compared to a more contoured region. They utilized 2D histograms from azimuth and elevation angles of each normal vector, formulating a ‘flatness score’ by counting the number of unidirectional vectors within predetermined bins, separately for left and right posterior regions of the head (Atmosukarto et al., 2010). Building on this foundation, Vuollo et al. refined the approach with KDE, a non‐parametric method for estimating the probability density function of a continuous random variable. This provides a more fluid representation of how normal vectors are distributed across the surface. The resulting flatness score quantifies the extent to which the normal vector distribution ‘deviates’ from a uniform distribution on a unit sphere (Vuollo et al., 2016).

**FIGURE 1:**
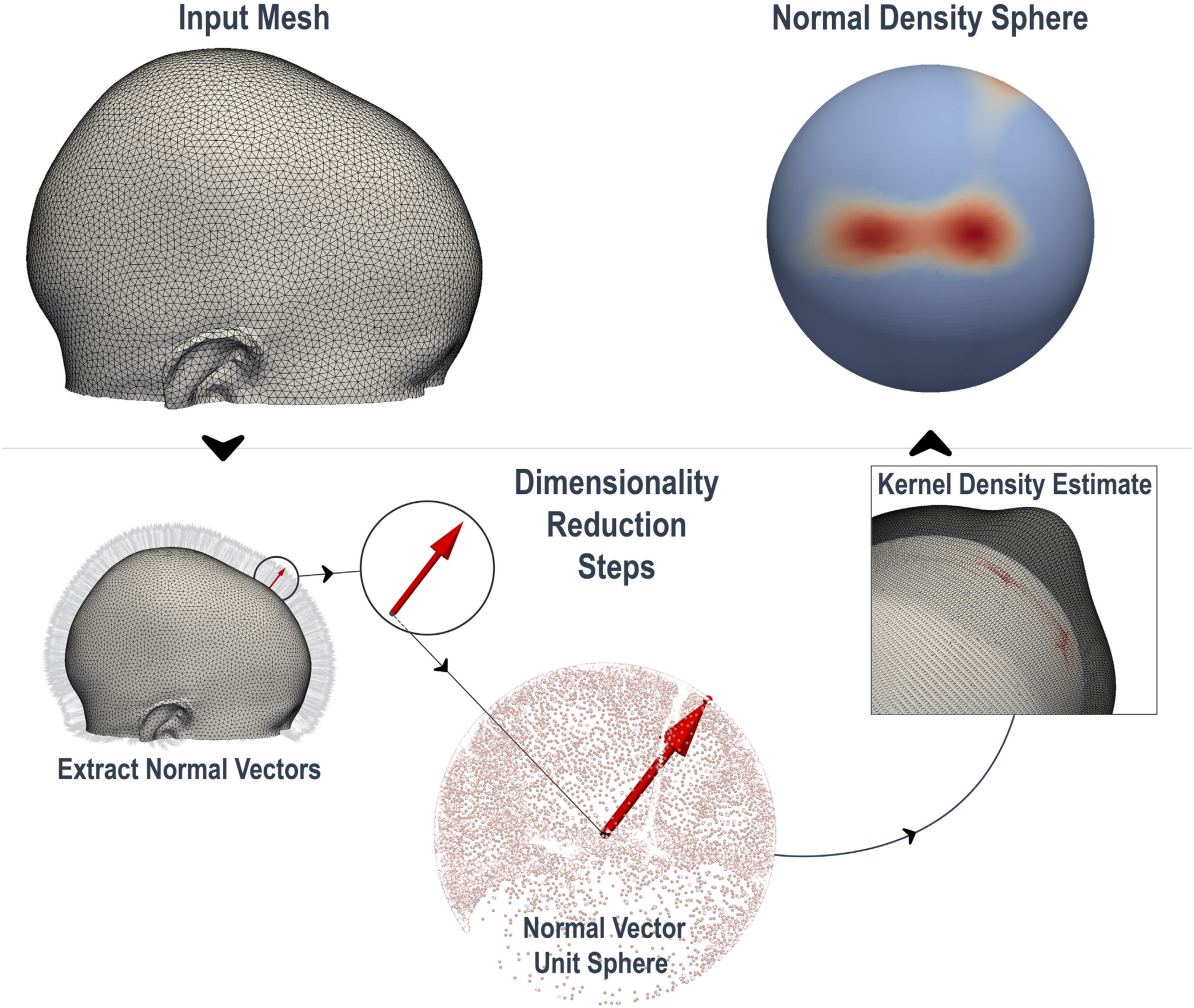
Dimensionality reduction steps; extracting the normal density sphere from a 3D mesh.

In pursuit of refining this shape‐based representation from 3D mesh data, our study further develops the KDE‐based shape analysis. The steps to convert a 3D image into its NDS, which integrate these principles, are described below and visualized in Figure 1.

By placing a kernel on each data point (normal vector direction) and summing the contributions from all these kernels we produce a continuous function from which the estimated normal vector density at a particular point can be obtained. We hypothesize that this array of density estimates captures morphological information that extends beyond mere flatness. Mathematically, our KDE function is represented as:

(1) $$\hat{f}\left(s\right)=\frac{1}{\mathrm{nh}}\sum_{i=1}^{n}K\left(\frac{s-{{\hat{N}}_{v}}_{i}}{h}\right)$$

Where:

- n is the number of data points (vertices)
- h is the bandwidth parameter which determines the smoothness of the estimated density function. A small value will fit the data more closely, while a large value will provide a smoother estimate. Scott's rule was used to determine the appropriate bandwidth (Scott, 2015).
- K is the kernel, which is a symmetric function that integrates to 1.
- s is any data point where the density needs to be estimated.
- ${{\hat{N}}_{v}}_{i}$ is the *i*th unit normal vector

For our application the kernel function is a Gaussian kernel, given by:

(2) $$K\left(u\right)=\frac{1}{\surd 2\pi }e^{-\frac{1}{2}u^{2}}$$

Where:

- u is the standardized distance between an observation and the point where density is being estimated. In our case, this standardized distance equals: $\frac{s-{{\hat{N}}_{v}}_{i}}{h}$

Integrating this Gaussian kernel (Equation 2) into our KDE function (Equation 1) gives us the density estimation at a data point s on the unit sphere as:

(3) $$\hat{f}\left(s\right)=\frac{1}{\mathrm{nh}}\sum_{i=1}^{n}\frac{1}{\surd 2\pi }e^{-\frac{1}{2}{\left(\frac{s-{{\hat{N}}_{v}}_{i}}{h}\right)}^{2}}$$

Higher values of $\hat{f}\left(s\right)$ (Equation 3) signal regions where vertex normals cluster closely, denoting predominant uniformity in direction or pronounced flatness. On the other hand, lower values are indicative of varied normal vectors in the surrounding geometry, suggesting a sparser or more intricate structure. This array of density values, when paired with its corresponding label (either normocephalic, trigonocephalic, or scaphocephalic), forms the foundational input data for our artificial neural network model.

### 2.3 Artificial neural network

**FIGURE 2:**
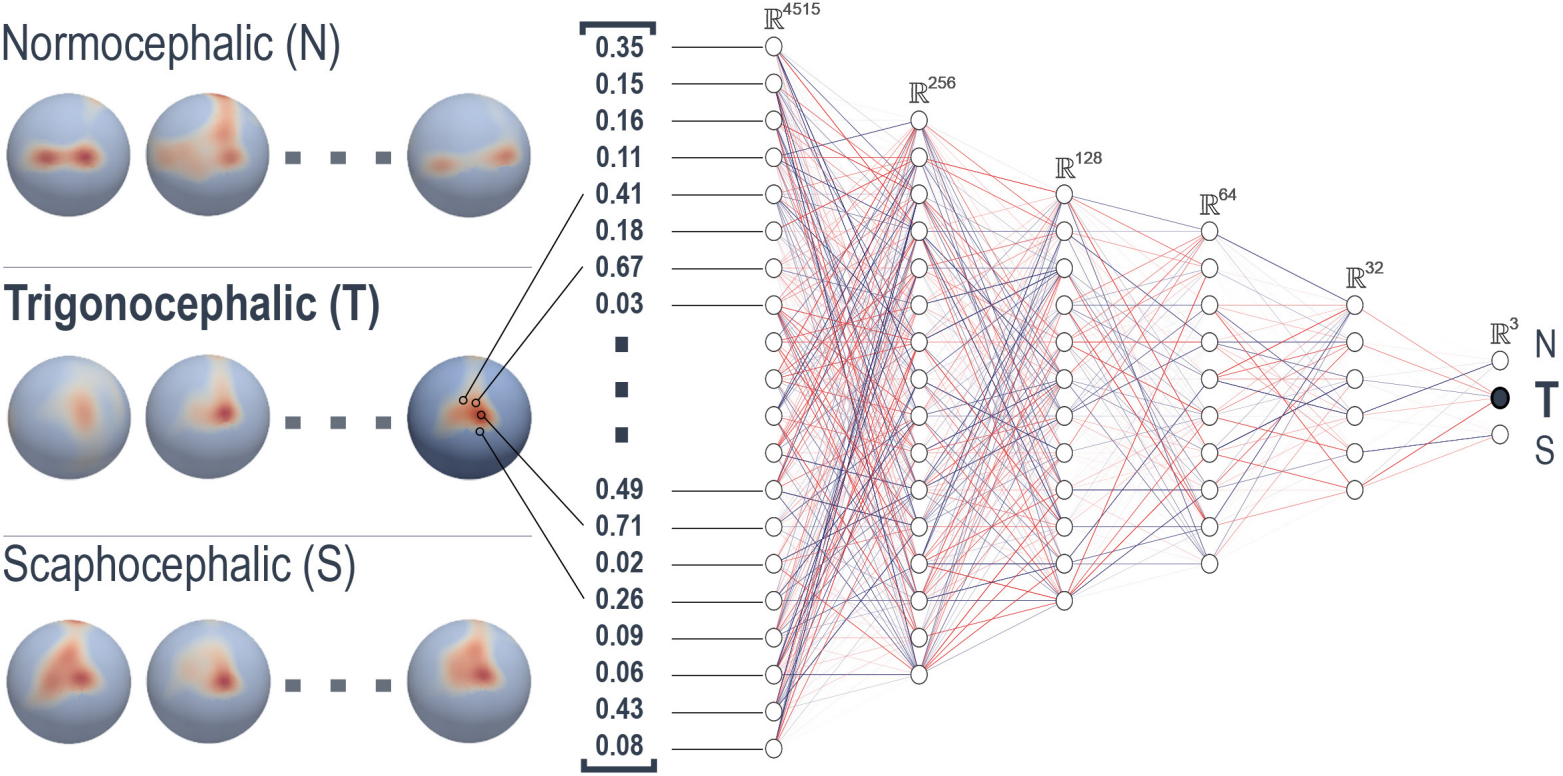
Fully connected neural network architecture utilized for training and analysis of normal density spheres.

The extracted array of density estimates served as the input to a 5‐layer, fully connected neural network. The architecture of this network, detailed in Figure 2, is structured with layers of dimensions 4515, 256, 128, 64, 32, leading to an output layer representing three classes: normocephalic, trigonocephalic, and scaphocephalic. A random 80/10/10 split was used to obtain data samples for training (80 normocephalic, 78 trigonocephalic, 82 scaphocephalic), validation (11 normocephalic, 9 trigonocephalic, 10 scaphocephalic), and testing (9 normocephalic, 13 trigonocephalic, 8 scaphocephalic), respectively.

From a technical perspective, the choice of a low‐dimensional representation offers several advantages. First, models trained on simpler representations tend to converge faster, reducing the overall training time. Additionally, by focusing on key features and reducing data complexity, the required sample size for achieving robust and generalizable models can often be reduced, mitigating potential overfitting and making the training process more efficient. Lastly, it leads to a significant reduction in the computational power required, making the analysis more feasible and efficient on a broader range of hardware platforms.

Prior to converting the outputs into probabilities for classification, the raw output value of the neural network (i.e., FP score) was compared with the original 3D image to assess if it can serve as a proxy for phenotypic severity. If so, an observable increase in overall phenotype should be observable in the 3D images at higher output values.

Attention maps were extracted, based on the computed Integrated Gradients, to pinpoint which features of the input array were of significance for each specific prediction (Sundararajan et al., 2017). By backpropagating through the neural network, this technique is used to pinpoint which input nodes contributed most substantially to the classification outcome. Specifically, these input nodes correspond to the KDE values, which are subsequently traced back to their originating normal vectors. Vital normal vector directions were then mapped to corresponding vertex points on the original 3D images—never processed by the network—to visualize and assess the anatomical regions that correspond to the extracted normal vector directions. Attention maps were generated and averaged across each class to display the most indicative nodes for the prediction of each phenotype. The red regions in the attention map indicate areas of high significance for the class prediction, whereas the blue regions represent areas of lower significance.

### 2.4 Expert severity scores

Two craniofacial experts (authors I.M.J. and M.V.V.) evaluated the overall phenotypic severity using a randomized subset of 60 synthetic head models. This included 3D data from both the trigonocephalic (*n* = 30) and scaphocephalic group (*n* = 30). A 4‐point scale for scoring was employed, where ‘0’ indicates normal, ‘1’ stands for mild, ‘2’ signifies moderate, and ‘3’ denotes severe, in accordance with published craniosynostosis photoscore guidelines (Gaillard, 2023a, 2023b). When there was a discrepancy in the scores between the two experts, the head model was excluded from the correlation analysis. The Spearman Rank Correlation Coefficient is used to determine the monotonic relationship between the computed FP score and (ordinal) clinical severity scores. A significance level less than 0·05 was considered significant for all statistical analysis.

## 3 RESULTS

### 3.1 Artificial neural network performance and attention maps

Our final model achieved 100% test accuracy. This means it was able to correctly classify every cranial class (normocephalic, trigonocephalic, scaphocephalic), solely based on the array of KDE values belonging to each of the head models. The complete process to classify and determine the severity of a single 3D image—encompassing the extraction of normal vectors, computation of the kernel density estimates, and prediction of the respective class and phenotypic severity—was accomplished in less than a second, averaging 0·753 seconds per sample (on CPU).

**FIGURE 3:**
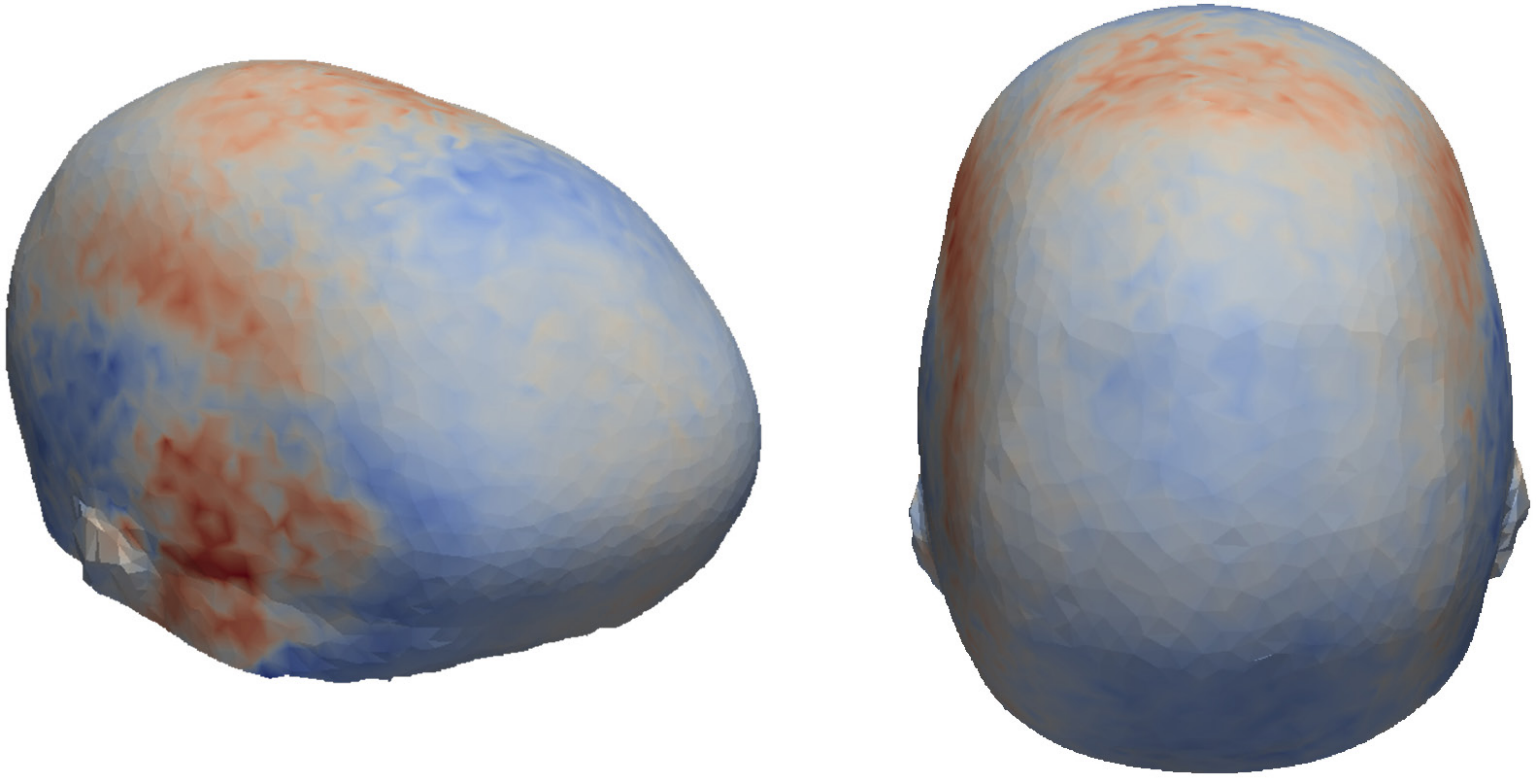
Neural network's average attention map for the scaphocephalic cranium.

**FIGURE 4:**
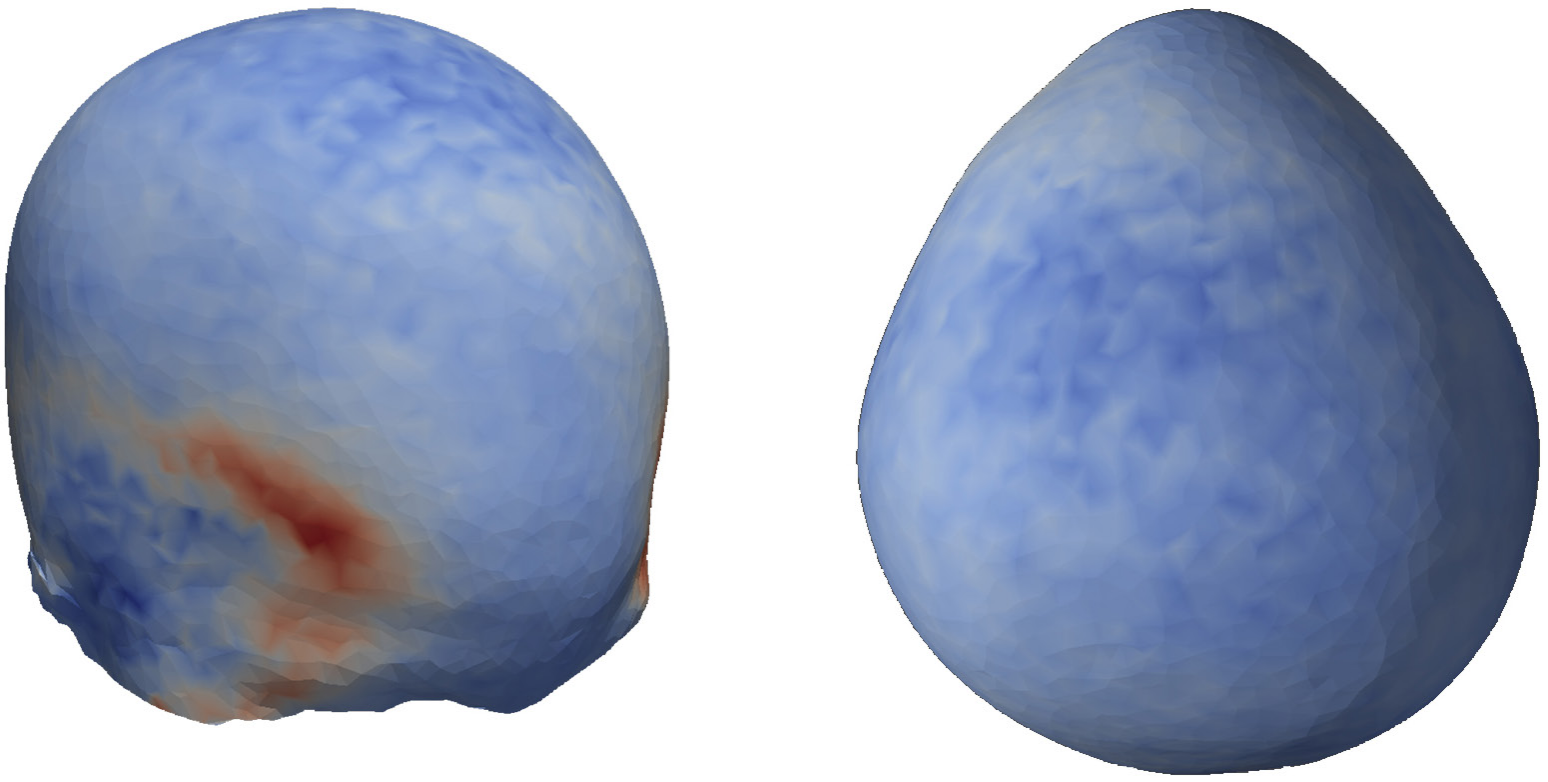
Neural network's average attention map for the trigonocephalic cranium.

In scaphocephaly, mean attention maps (Figure 3) show that the network considered normal vector directions associated with the parietal and temporal bones, as well as those indicative of vertex depression, most important. Slightly less critical, yet still important were vectors related to occipital pinching and frontal bossing. In trigonocephaly, mean attention maps (Figure 4) show that most of the important features are located on and around the temples, likely related to the severity of temporal hollowing.

In head models with less prominent phenotypes (i.e., lower FP score), network attention visibly shifts. In both scaphocephalic and trigonocephalic head models, a reduction in network attention for the temples was observed. This attention is redirected toward the occiput in scaphocephalic models, and toward the metopic ridge and vertex in trigonocephalic models.

Random rotations, translations, and uniform scaling applied to the input mesh, prior to extracting its NDS, resulted in no change in the computed FP severity score and attention maps, as visualized in Supplementary Figure S1.

### 3.2 The feature prominence score as a severity proxy

A set of 60 3D head models, comprising 30 scaphocephalic and 30 trigonocephalic models, was assessed by two craniofacial experts. The 3D models provided to the experts could be translated and rotated for optimal viewing. Out of these 60 head models, consensus was achieved for 28 scaphocephalic and 26 trigonocephalic models. The primary objective was to evaluate the existence and magnitude of a positive monotonic relationship between the FP score and clinical severity scores.

**FIGURE 5:**
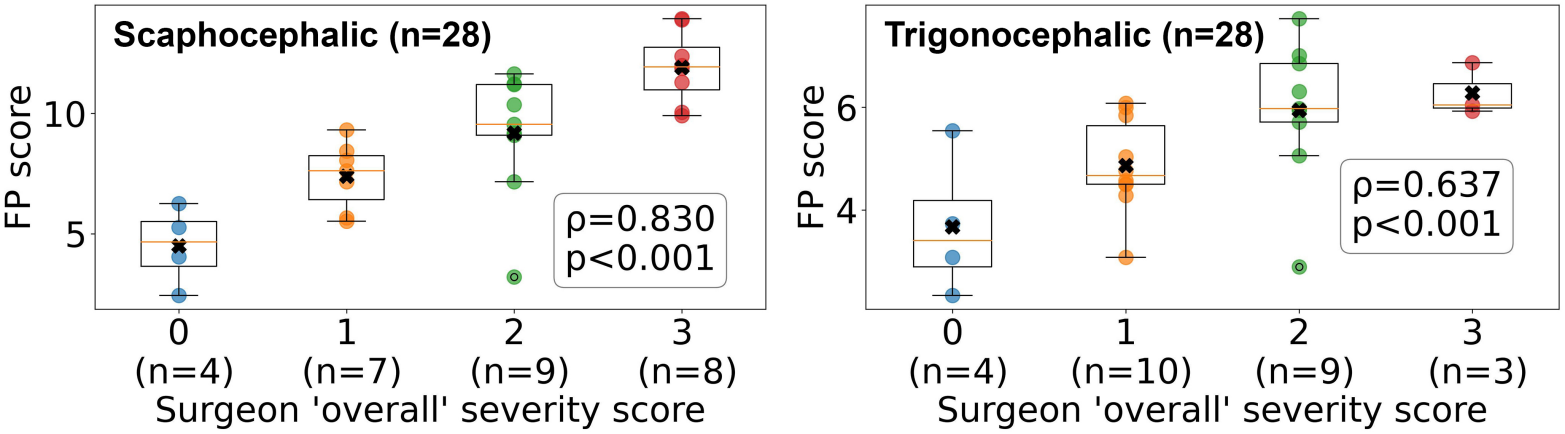
Box plot of computed Feature Prominence scores from scaphocephalic (left) and trigonocephalic (right) head models versus clinical severity scores: normal (0), mild (1), moderate (2), and severe (3). Spearman's rank correlation coefficient: 0.83 (scaphocephalic) and 0.64 (trigonocephalic).

Utilizing the Spearman Rank Correlation Coefficient, a very strong positive monotonic relationship was identified for the scaphocephalic models (*ρ* = 0.83, *p* < 0.001), and a strong relationship was observed for the trigonocephalic models (*ρ* = 0.64, *p* < 0.001). These relationships were both statistically significant and are visualized in Figure 5.

**FIGURE 6:**
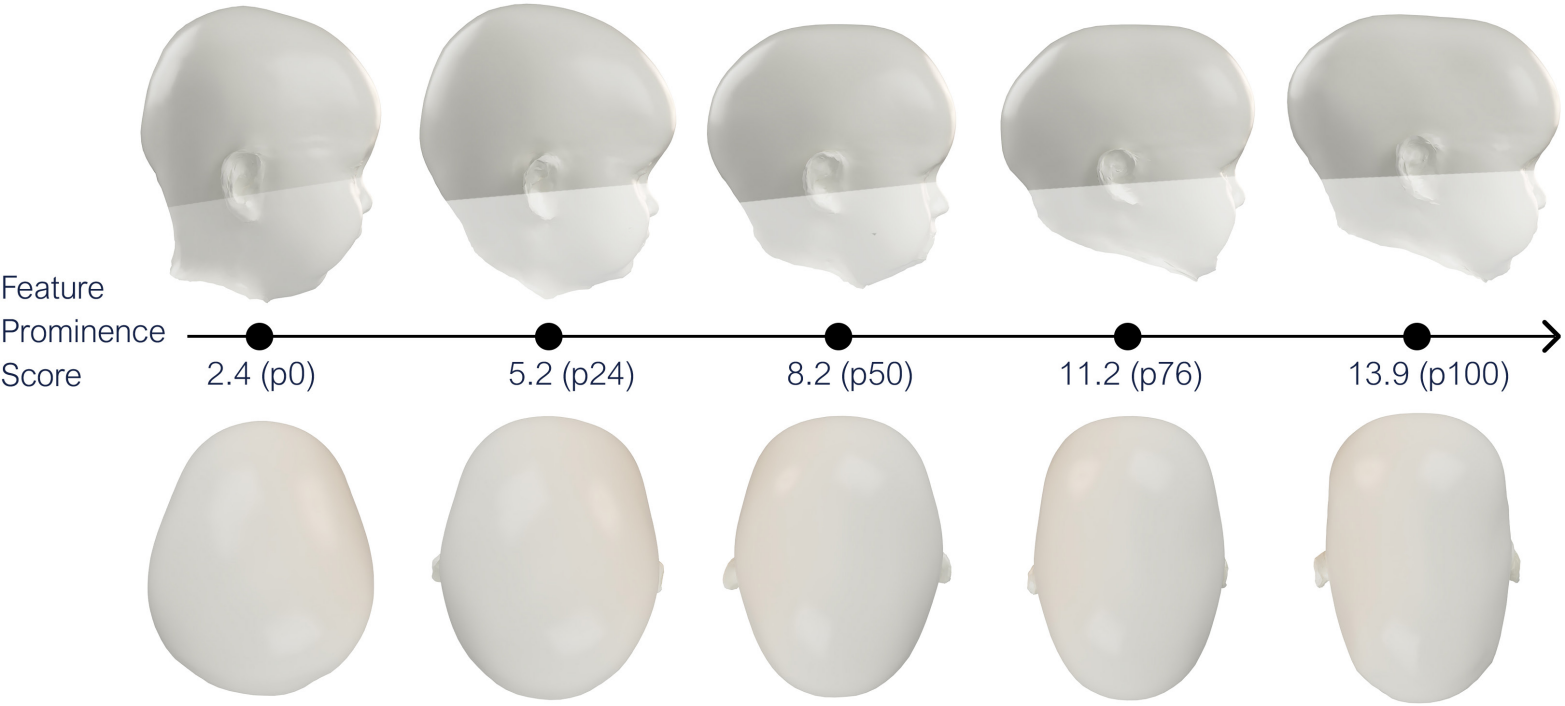
Relationship between the FP score (neural network output values) and their corresponding scaphocephalic head model.

**FIGURE 7:**
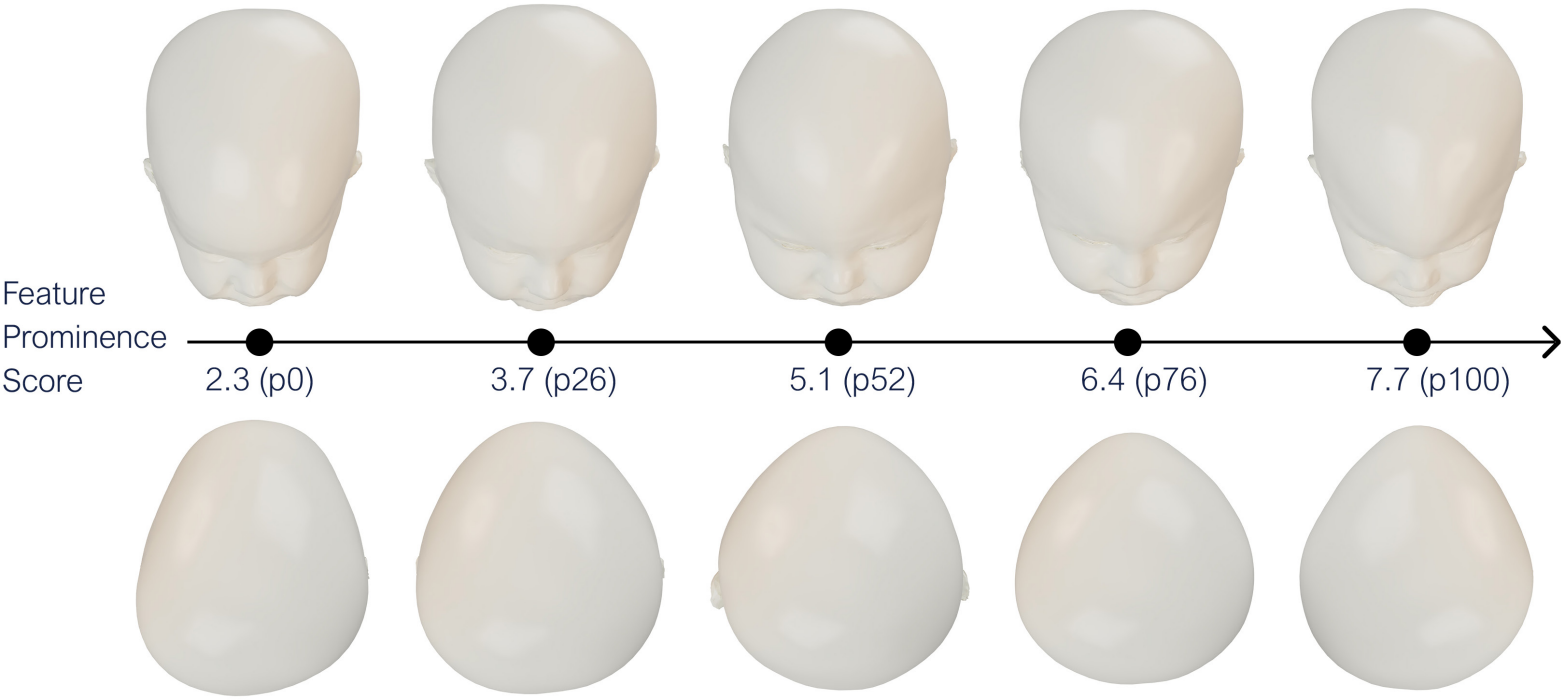
Relationship between the FP score (neural network output values) and their corresponding trigonocephalic head model.

To offer a visual representation, 3D head models from the dataset whose FP score values were nearest to the 0th, 25th, 50th, 75th, and 100th percentiles were selected. The scaphocephalic and trigonocephalic head models are presented in Figures 6 and 7 respectively. It can be observed that as the FP value rises, the phenotypic severity becomes increasingly evident.

## 4 DISCUSSION

In this study, we demonstrate that the density distribution of normal vectors embeds critical information about cranial shape. When using this low‐dimensional shape representation in combination with an artificial neural network, it becomes feasible not only to classify cranial shapes but also to use the raw output values of the network as a proxy for the severity of related phenotypes in relation to the reference (training) dataset, introducing the FP score. Although our focus is primarily on cranial dysmorphologies, the potential applications of the FP score stretch to other fields like biology, anthropology, and morphogenesis in various organisms.

To compute the KDE on a sphere, various methods suitable for spherical data, such as the Von Mises‐Fisher distribution, have been proposed. However, empirical results have shown that our trained models using the Gaussian kernel consistently outperformed the Von Mises‐Fisher distribution. This might seem counterintuitive, but we hypothesize that due to the small distances between point normals—especially within distinct clustered regions associated with each subtype—the distortion effect is minimal. Additionally, since the network is trained using data processed with Gaussian KDE, it may effectively interpret the most essential density information and compensate for any minor inaccuracies introduced by Euclidean assumptions. In our context, density patterns associated with specific pathological features are more critical than maintaining precise angular relationships, which methods for spherical data are able to capture more accurately. This could unnecessarily complicate the learning task, potentially explaining the quicker convergence and improved performance with Gaussian KDE.

Recognizing the potential of the proposed methodology in broader contexts, where angular relationships might be more significant, we have retained the Von Mises‐Fisher kernel as an optional parameter in the source code. This allows users to explore its application when training their own models. Readers interested in a more comprehensive mathematical reference are referred to earlier works discussing the structure and features of spherical data residing on the unit sphere in 3D space (Vuollo & Holmström, 2018).

The utilized network is fundamentally a classification model, trained to categorize to which cranial shape class a given KDE array belongs. The network's performance showed that the network is able to perform this task consistently. Importantly, no signs of overfitting were observed.

The proposed low‐dimensional representation captures essential shape characteristics without biases introduced by geometric size or spatial orientation. This was achieved by combining non‐rigid registration techniques that fix the mesh topology, with the proposed KDE technique. This allows for shape‐based comparisons in a longitudinal setup without the need to correct for normal growth or set reference points for registration. Unlike utilizing coordinate positions or normal vectors—both of which are directly influenced by changes in size and spatial orientation—the KDE values remain constant. Consequently, the application of random rotations, translations, and uniform scaling to the input mesh did not affect the outcomes, both in terms of the FP score value and attention map (Supplementary Figure S1).

Both the KDE array and the normal density sphere representation (from which the KDE values are derived) ensure complete patient anonymity. This addresses a frequent challenge in multi‐center collaborations where patient data sharing is essential. Moreover, employing KDE offers computational advantages. Not all craniofacial centers possess the computational resources required to train geometric deep learning models on extensive 3D datasets effectively. However, with our approach, they can still leverage the power of machine learning methods to objectively analyze the complex cranial morphology.

Considerable efforts in the field related to explainable AI are being made to enhance model interpretability. In this study, we used integrated gradients to derive which input features were of importance to the network and averaged the resulting attention maps for each class individually (Figure 4) (Sundararajan et al., 2017).

The utilization of attention maps serve two key features:

1. They validate the network's decision‐making process, affirming its alignment with known phenotypes associated with craniosynostosis subtypes.
2. They aid in the interpretation of the results. Regions highlighted for their importance in making a prediction, directly influence the severity metric value. Therefore, the severity metric is most indicative of the phenotypes that are described by variations in these regions.

Our results showed that regions of interest to the network correspond with anatomical regions that are associated to the different craniosynostosis subtypes and therefore clinically relevant. An interesting observation included the shift in attention as the FP score changed. This may be indicative of the network's adaptability in recognizing varying severity levels and underscores the complexity of the cranial features related to craniosynostosis. However, quantitative evaluations of these interpretability methods are difficult due to a lack of ground truth, particularly in our case where the 3D shape characteristics are embedded within a one‐dimensional representation.

Previous studies have demonstrated that normal vectors can be used to identify and quantify regions of increased flatness (Atmosukarto et al., 2010; Vuollo et al., 2016). This is particularly relevant to the scaphocephalic phenotype, characterized by an elongated cranium where the parietal bones and the flattened vertex result in a predominance of certain normal vector orientations. However, the trigonocephalic phenotype, which includes features like temporal hollowing and varying degrees of forehead wedging, produces complex curvatures. These complexities translate into intricate patterns on the normal density sphere, challenging to identify without advanced methods. Notably, the model was not only able to classify trigonocephaly but also accurately quantify its phenotypic severity based on the relevant anatomical regions, as indicated by the attention maps in Figure 4. These attention maps suggest that the network is comprehensively considering the normal vector directions corresponding to the regions of interest.

In the clinical assessment of 60 3D head models, two craniofacial experts achieved consensus on 54 models. They used published guidelines on photoscores for both scaphocephaly and trigonocephaly during their assessment (Gaillard, 2023a, 2023b). These guidelines dictate that images should be scored based on selected phenotypical characteristics and overall phenotypical severity. However, the agreement on phenotypical characteristics was deemed suboptimal, whereas there was substantial agreement on overall phenotypic severity scores. Therefore, in this study, we limited the assessment to overall phenotypic severity scores. If there was a discrepancy in scores between the two experts, that particular image was excluded from the correlation analysis. While these challenging boundary cases underscore the need for a continuous and objective metric, they could not be utilized to validate the newly introduced FP severity score.

During validation, we investigated both the presence and intensity of a positive monotonic relationship between the FP score and the clinical severity scores. Using the Spearman Rank Correlation Coefficient, a very strong positive monotonic relationship was found for the scaphocephalic models (ρ = 0.83, *p* < 0.001) and a strong relationship for the trigonocephalic models (ρ = 0.64, *p* < 0.001). This relationship is visualized in Figure 5, showing a clear increase in the mean FP score as the clinical severity scores given by the surgeons increase.

The absolute value of the FP score is influenced by both the training dataset and network architecture. Therefore, we derived a percentile‐based normalized scale from these raw values. This also means that with a different training set, the same percentile might equate to a different phenotypic severity. This consideration partially influenced our decision to use a public dataset for demonstrating the proposed methodology, ensuring both reproducibility and benchmarking. Based on the computed percentiles, the 0th, 25th, 50th, 75th, and 100th percentile scaphocephalic and trigonocephalic models are illustrated in Figures 6 and 7, respectively. These figures clearly show how a significant rise in the FP score corresponds to a noticeable increase in observable phenotypic severity. Combined with the results from Figure 5, we are confident that the FP score effectively captures clinically relevant features necessary for assessing phenotypic severity in both scaphocephaly and trigonocephaly.

**FIGURE 8:**
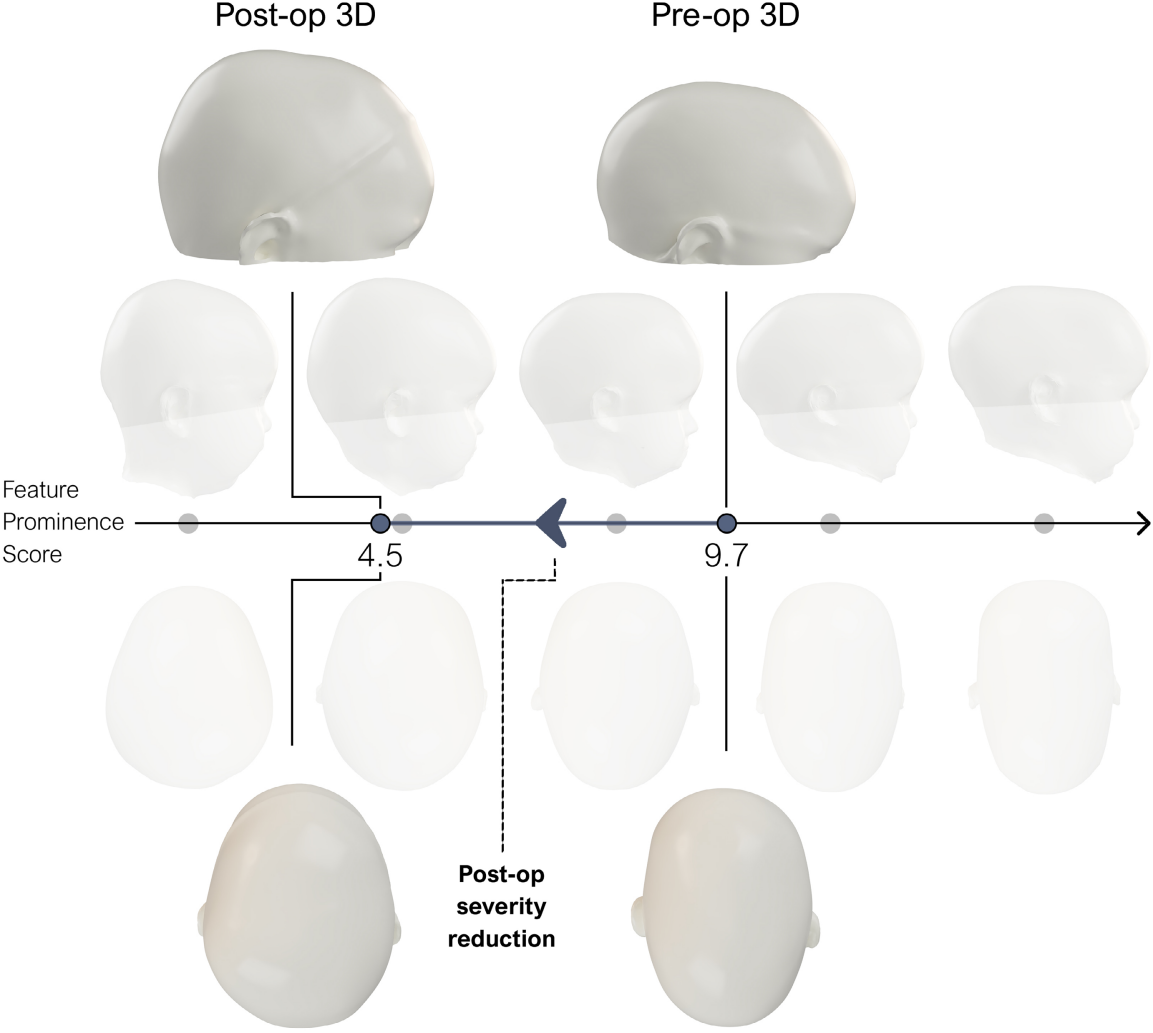
Patient (male) treated using spring‐assisted cranioplasty at the age of 6 months and compared to the post‐operative 3D image acquired at the age of 13 months. Figure illustrates surgical effect with pre‐and post‐operative severity scores 9.7 and 4.5, respectively, corresponding to a 45% cranial shape improvement.

Future work includes validation of this metric with clinical data and preparing for its evaluation in a multi‐center context. This validation will enable the assessment of phenotypic severity pre‐ and post‐surgery, thereby offering objective insights into the efficacy of surgical interventions. For instance, Figure 8 illustrates the measurable effects of spring‐assisted cranioplasty on a male patient at the age of 6 months, demonstrating a significant improvement in cranial shape from a pre‐operative FP score of 9.7 to a post‐operative FP score of 4.5, which indicates a 45% improvement. However, the effect of noise inherent to clinical data upon the FP scores requires further investigation and extends beyond the scope of this initial proof‐of‐concept study, which demonstrates the theoretical capabilities of the proposed methodology under controlled conditions with synthetic data. The utilized synthetic data generated from a statistical shape model ensures that the shape variations are (statistically) well‐defined and consistent with the known characteristics of these conditions through weighted principal component analysis.

Challenges that are faced when using raw patient data include surface artifacts. Frequently observed artifacts include protrusions due to hair that is tucked underneath a hair cap during acquisition. Despite this, the unique design of our neural network, which is trained on a low‐dimensional shape representation that utilizes KDE values derived from a probability density function, is expected to be robust against such issues. By applying the KDE method with a suitable bandwidth, we can approximate a smooth function that reflects the underlying density of the data. This approach inherently reduces the impact of outliers, consequently making the network more resistant to distortions introduced by surface artifacts. This is another key advantage of our low‐dimensional representation approach and suggests that the network should maintain high performance even when confronted with the complexities of raw patient data.

Considering mesh topology is crucial when applying the proposed method to different datasets. Adaptive mesh refinement, commonly used in 3D systems, improves reconstruction quality by increasing mesh density in areas of high curvature, leading to anisotropic meshes. Yet, if we want the distribution of normal vectors to embed global phenotypic patterns, this anisotropic mesh property can introduce biases, especially in complex areas like ears versus smoother areas such as head sides where the parietal bones lie beneath. We therefore recommend to verify the distribution of mesh elements across the 3D surface prior to extracting its NDS. If the mesh topology is inconsistent across the dataset, resampling may be required.

In conclusion, the FP score is a novel and easy to implement metric that can be widely adopted to evaluate and compare treatments across different centers, facilitating large multi‐center studies needed to reveal new insights into the effect of different treatments on the cranial shape. Our group's participation in the ERN CRANIO network, a large European consortium on rare diseases, highlights the recurrent challenges with data sharing, particularly with privacy‐sensitive 3D cranial data, for which our FP score could offer a significant resolution. Furthermore, this technique surpasses traditional measurements by providing the capacity for a detailed analysis of 3D morphology. This advancement is a leap toward meeting the need for both national and international collaborative work. To further aid in this collaborative effort, and to enable other institutions and consortia in adopting this metric within their research endeavors, the source code used for this study was made publicly available upon publication (https://github.com/T‐AbdelAlim/FPscore).

## 5 CONCLUSION

In this study, we have shown how the distribution of normal vectors can innovatively quantify cranial shape. We have combined a low‐dimensional representation with neural networks to classify cranial shapes and introduce the FP score, a novel quantitative metric for phenotypic severity.

Our method offers an accessible AI‐based approach with key advantages: it removes size bias, is unaffected by spatial orientation, preserves patient anonymity for multi‐center studies, and due to its efficient representation, is accessible to craniofacial centers regardless of computational resources, with the source code publicly accessible for ease of implementation.

Attention maps have made our model more interpretable and show that the network's attention is directed at regions associated with distinct craniosynostosis phenotypes, while the FP score's correlation with clinical severity scores confirms its robustness and relevance. We aim to validate the model on larger patient datasets and anticipate extending its applicability beyond cranial shape analysis. The adoption of this metric promises to refine clinical decision‐making and enable multi‐center collaborations without sacrificing data privacy, thus progressing patient care and advancing the scope of craniofacial research.

## AUTHOR CONTRIBUTIONS

Concept/design: Tareq Abdel‐Alim, Marie‐Lise van Veelen, Gennady Roshchupkin. Acquisition of data: Tareq Abdel‐Alim, Marie‐Lise van Veelen, Irene Mathijssen. Data analysis/interpretation: Tareq Abdel‐Alim, Franz Tapia Chaca, Gennady Roshchupkin, Marie‐Lise van Veelen, Irene Mathijssen. Drafting of the manuscript: Tareq Abdel‐Alim, Marie‐Lise van Veelen, Gennady Roshchupkin. Critical revision and approval of the manuscript: Clemens Dirven, Wiro Niessen, Eppo Wolvius, Irene Mathijssen.

### OPEN RESEARCH BADGES

This article has earned an Open Materials badge for making publicly available the components of the research methodology needed to reproduce the reported procedure and analysis. All materials are available at https://github.com/T‐AbdelAlim/FPscore (For an example of our commitment to open‐source resources, please refer to our previous repositories at https://github.com/T‐AbdelAlim/CraniumPy and https://github.com/T‐AbdelAlim/LBOmeshfilter).

## Supporting information


Figure S1:

## Data Availability

The data that support the findings of this study are openly available on Zenodo: “A statistical shape model of craniosynostosis patients and 100 model instances of each pathology” at https://zenodo.org/records/6390158, reference number (DOI) https://doi.org/10.5281/zenodo.5638147.
