# Supplementary material for: Quantifying dysmorphologies of the neurocranium using artificial neural networks
Source: J Anat. 2024 May 17;245(6):903–13. doi: 10.1111/joa.14061 (PMC11547242; doi:10.1111/joa.14061)
Supplement: Supplementary file 1 — Figure S1: [file JOA-245-903-s001.docx]

**Supplementary Figure 1:** **Comparing the original mesh (left) with its rotated, translated, and uniformly scaled counterpart (right) reveals consistent NN attention maps and identical FP scores of 8.4.**

**
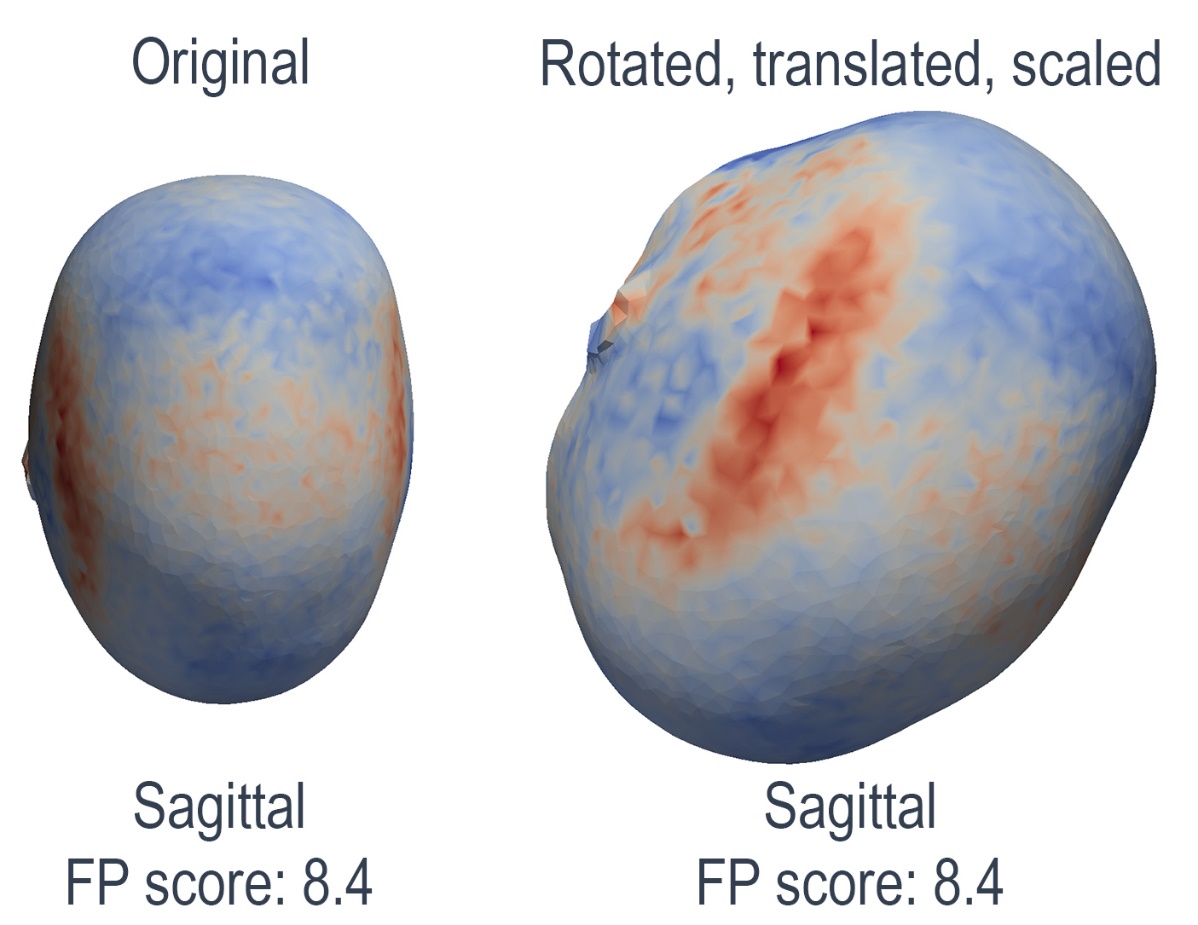
**
